# The Effect of Laparoscopic Dermoid Cystectomy on Anti-Mullerian Hormone: A Case Series

**DOI:** 10.14740/jmc5276

**Published:** 2026-04-29

**Authors:** Praneel Kumar, Harry Merkur, Peter Russell, Clare Wong

**Affiliations:** a Department of Obstetrics and Gynaecology, Blacktown Hospital, Sydney, Australia; b Adelaide Medical School, Faculty of Health and Medical Sciences, Adelaide University, Adelaide, Australia; c Faculty of Medicine, Western Sydney University, Campbelltown, Australia; d Department of Anatomical Pathology, Douglass Hanly Moir Pathology, Macquarie Park, Australia

**Keywords:** Dermoid cyst, Fertility, Laparoscopy

## Abstract

Laparoscopic cystectomy for benign ovarian pathologies overall has been shown to reduce anti-Mullerian hormone (AMH), an important biomarker of ovarian reserve and fertility; however, the effect of dermoid cystectomy on ovarian reserve remains unclear. We sought to measure the change in AMH from before and after planned laparoscopic dermoid cystectomy at our surgical center in a case series. Females of reproductive age between 18 and 40 years presenting with a pre-operative diagnosis of a unilateral dermoid cyst on pelvic ultrasound and an indication for laparoscopic cystectomy were recruited. AMH levels were measured pre-operatively and repeated post-operatively between 6 and 8 weeks and the histology was checked to confirm a dermoid cyst. The laparoscopic surgery was undertaken using a standard technique at our center by senior gynecologists. A non-parametric Wilcoxon signed-rank test was performed to assess the difference between paired serum AMH samples. Of the seven females with dermoid cyst on final histopathology, there was no statistically significant difference observed between the pre-operative and post-operative AMH levels (W-value 13, P-value not less than 0.05). Our case series did not show any difference between pre-operative and post-operative AMH results following laparoscopic dermoid cystectomy and more data are required on the impact on ovarian reserve from future studies.

## Introduction

Dermoid cysts, also called mature cystic teratomas, are a benign ovarian cyst and account for 70% of ovarian cysts in females aged less than 30 years of age [1]. Dermoid cysts that cause abdominal pain or enlarge are recommended for surgical removal. Laparoscopic cystectomy, where available, is the standard of care given the advantages of minimally invasive surgery such as faster recovery time, reduced post-operative pain, and improved cosmesis [2, 3].

The effect on fertility is an important consideration when performing ovarian surgery on reproductive aged females. Characteristics of the cyst such as endometrioma histology, increased size, and bilaterality have been shown to decrease ovarian reserve following cystectomy [2–8]. Injury to the ovary resulting from cystectomy can also occur and decrease ovarian reserve [4, 9] which can be more pronounced with certain surgical techniques such as the use of electrosurgery for hemostasis [2, 8, 10].

Current studies use a variety of measures to assess the effect of cystectomy on ovarian reserve. Anti-Mullerian hormone (AMH) is released by early-stage follicles in the ovary and levels decrease with increasing age [2, 11]. AMH is a commonly used biomarker given that it is simple to collect with venepuncture and levels remain stable during the menstrual cycle [2]. Studies measure AMH following cystectomy at different time points, ranging from 1 day post-operatively to 12 months [9, 12, 13]. Other assessments of ovarian reserve include follicle stimulating hormone levels, pelvic ultrasound (for ovarian volume, Doppler, and antral follicle counts), histopathology specimens for follicle counts, and *in-vitro* fertilization (IVF) cycle success rates [1, 4, 5, 12, 14].

The current evidence on the effect of dermoid cystectomy on ovarian reserve is lacking. A meta-analysis of the excision of non-endometriotic benign cysts, which included dermoid cysts, showed that cystectomy decreased serum AMH levels by a mean of 1.14 ng/mL (95% confidence interval (CI) −1.36, −0.92) (equivalent to 8.1 pmol/L) over 1 to 6 months [11]. However, by combining all benign cysts in a single group, the effect of dermoid cystectomy on ovarian reserve cannot be distinguished. Primary studies also group dermoid cystectomies with other benign pathology and dermoid cysts are often in the minority within the benign pathology grouping [5, 6, 12, 14–16]. The handful of studies that separately report the effect of dermoid cystectomy on ovarian reserve have relatively small sample sizes (ranging from 7 to 33) [7–9, 17, 18]. These studies also show conflicting results. For example, Lind et al reported a 25% reduction in AMH levels 6 months following dermoid cystectomy [17], whereas Henes et al did not detect a change from pre- to post-operative AMH levels [7]. Amooee et al reported that declines in AMH levels were greatest with dermoid cysts compared to mucinous cystadenomas and serous cystadenomas [18].

The effect of dermoid cystectomy on ovarian reserve merits further investigation as they are distinct from other benign ovarian cysts. Dermoid cysts contain inflammatory material and spillage of the cyst contents during surgery can result in chemical peritonitis, which may affect ovarian function [1]. Dermoid cysts are also relatively common and information relating to the effect of dermoid cystectomy on fertility can aid in the counseling of patients planned for surgery [1]. To understand the effect of planned laparoscopic dermoid cystectomy on ovarian reserve, we measured the change in serum AMH before and after surgery in a small case series. Our secondary aim was to record any ovarian follicles observed on the histopathology specimen after excision of the dermoid cyst.

## Case Report

### Methods

#### Study design

We performed a study where participants acted as their own controls given the pre-test post-test design. Our study was concerned with measuring changes in AMH levels within a participant as opposed to between participants. Clinical differences between participants are presented for completeness in the case series.

#### Study participants

Females aged 18–40 years, referred to the gynecology clinic with a suspected dermoid cyst on pelvic ultrasound and requiring laparoscopic ovarian cystectomy, were eligible. Indications for surgery included symptoms due to the ovarian cyst, such as pelvic pain, and persistence or enlargement of the ovarian cyst. Exclusion criteria included suspicion of malignancy or previous malignancy, non-dermoid cyst on pelvic ultrasound and/or final histopathology, previous diagnosis of infertility of any cause, changes in hormonal medication use that could affect AMH measurements from participant enrollment to their post-operative AMH measurement, pituitary, thyroid or adrenal disorders and pregnancy. No participants had taken oral contraceptive pills within 3 months of the study enrollment or the AMH measurements.

#### Surgical procedure

Specialist gynecologists with training in minimally invasive surgery at the same center performed the operations. All participants were consented for a unilateral laparoscopic ovarian cystectomy. Following general anesthesia, an umbilical incision was made for abdominal entry, pneumoperitoneum was established, and a 10 mm umbilical trocar inserted. Two accessory trocars were placed under direct laparoscopic vision in the lower abdominal quadrant. Using monopolar scissors, the ovarian cyst was incised, the capsule identified, and the cyst removed using traction with laparoscopic forceps, taking care to preserve the ovarian parenchyma and avoiding spillage of the cyst contents. Hemostasis was achieved with sutures, diathermy, or sealants. Conversion to laparotomy was performed in an emergency or if the cyst could not be removed laparoscopically. Following surgery, the participants were observed for a maximum of 48 h and then discharged.

#### Outcome measurement

Venepuncture was used to collect a pre-operative (baseline) and post-operative serum AMH sample, regardless of the menstrual cycle day. The pre-operative serum AMH was collected within 4 months of surgery from all participants. The post-operative sample was collected between 6 and 8 weeks following surgery from all participants. This timeframe allowed for inflammation to have resolved post-surgery. The samples for each participant were tested using the same immunoassay method at the central laboratory, which had a lower limit of quantitation of 1 pmol/L and an inter-assay coefficient of variation of less than 5.6%. The excised ovarian cyst was reviewed on histopathology to confirm a dermoid cyst, and the presence of any primordial follicles observed by a qualified pathologist in the histopathology report was recorded. The presence of primordial follicles was not a quantitative assessment.

#### Statistical analysis

The paired pre-operative and post-operative serum AMH concentrations for each participant were compared using a non-parametric, two-tailed, Wilcoxon’s signed-rank test. The test is appropriate for dependent samples and small sample sizes. A P-value less than 0.05 was considered statistically significant. Statistical analysis was performed using Stata (StataCorp, Texas, USA, version 18.5).

#### Ethical approval

All participants provided written informed consent prior to enrollment. The study was approved by the Western Sydney Local Health District Human Research Ethics Committee (approval number 2020/ETH02973) and followed all ethical guidelines.

### Results

We recruited 10 participants for our study; however, two were excluded from the final analysis as they had a non-dermoid cyst on final histopathology and one participant did not complete their post-operative AMH measurement.

The seven participants in our case series had an age range from 18 to 40 years with a unilateral dermoid cyst, varying in size from 4 to 8 cm (see Table 1 for case information). Five participants were nulliparous.

**Table 1:** Participant Case Details

Case ID	Age, years	Cyst size, cm	Surgery indication	Parity	Operative details	Hemostatic method	Preop AMH, pmol/L	Postop AMH, pmol/L	Primordial follicles on histopathology
1	24	4	Persistent cyst and pelvic pain	P0	Nil spillage	Suturing	37.9	39.8	Yes
2	40	8	Pelvic pain	P0	Converted to laparotomy due to large cyst size	Suturing	2.2	<1.0	Yes
3	18	8	Pelvic pain	P0	Ruptured cyst	Suturing	39.6	49.8	No
4	27	4	Persistent cyst	P1	Ruptured cyst	Diathermy	17.7	14.2	Yes
5	36	10	Persistent cyst	P0	Ruptured cyst	Not required	12.2	13.9	No
6	28	5	Pelvic pain	P2	Ruptured cyst	Hemostatic sealent (surgicel)	30.4	31.8	No
7	27	7	Persistent cyst and pelvic pain	P0	Nil spillage	Suturing	22.6	14.5	No

Cyst size in cm as per operative or histopathology report. AMH: anti-Mullerian hormone; Preop: pre-operative; postop: post-operative.

The main indication for surgery was pelvic pain (n = 5/7). Four participants had spillage of the dermoid cyst intra-operatively. One laparoscopic surgery was converted to open surgery due to difficulty removing the dermoid cyst while avoiding spillage. Diathermy for hemostasis was used in only one participant. There were no post-operative complications noted.

There was no statistically significant difference between the pre-operative AMH and post-operative AMH values (Wilcoxon’s signed-rank test P-value 0.9375). Three participants had histopathology samples noting the presence of primordial follicles.

## Discussion

Our case series did not find a statistically significant difference in females of reproductive age planned for laparoscopic dermoid cystectomy in pre- to post-operative serum AMH levels. This is a similar finding to Henes et al study (n = 7) [7] that did not detect a difference in AMH levels following dermoid cystectomy, however, differs from several other studies, including a systematic review of all benign non-endometriotic ovarian cysts (n = 367) and dermoid cysts (n = 23), respectively [11, 18]. Histopathology reports noted the presence of primordial follicles in only three out of the seven participants.

Inferring the impact of dermoid cystectomies on ovarian reserve is currently required given the paucity of studies investigating this effect directly. Evidence from a clinical trial which compared different methods of hemostasis during dermoid cystectomy can be informative [13]. Liang et al (n = 80) reported a statistically significant decrease, ranging from 16% to 20% in serum AMH levels at 12 months after endoscopic dermoid cystectomy compared with pre-operative levels, irrespective of the hemostasis method used [13]. This would suggest that dermoid cystectomy reduces ovarian reserve similar to other benign ovarian cystectomies. Huang et al study (n = 71) which investigated single-port versus conventional laparoscopy for dermoid cystectomy reported a 15% decrease from pre-operative to post-operative AMH levels at 4 weeks in both arms [19].

When a decrease in serum AMH has been detected post-operatively, several studies have found recovery. Ding et al (n = 90) reported that while serum AMH levels decreased following laparoscopic cystectomy for benign ovarian cysts at 1 month, there was no difference at 6 and 12 months when compared to pre-operative levels [12]. Similar findings have also been replicated by Li et al (n = 56) [20] at 6 months. Chang et al (n = 20) [5] and Amooee et al (n = 23) [18] reported AMH recovery as soon as 3 months post-cystectomy. While the recovery of AMH levels is not a universal finding in the current literature [13], these results can provide some reassurance on the effect of fertility during patient counseling. Similarly, serum FSH levels following benign non-endometriotic cystectomies have also been reported to remain stable from baseline to 6 months post-operatively [4, 12, 20].

Strengths of our study include its prospective design, adherence to standardized operative technique, and measurement of AMH levels using the same assay method for each participant. Our study also provides data specific for dermoid cysts, which are not consistently reported in the current literature. It can therefore inform future systematic reviews and meta-analysis on this topic.

While our case series cannot provide definitive evidence on the effect of laparoscopic dermoid cystectomy on ovarian reserve due to the small sample size, it can inform future studies. For example, we only measured one marker of ovarian reserve post-operatively at one point in time, and changes in AMH levels may also not indicate changes in fertility levels, therefore additional markers could be considered in future studies. Moreover, there is significant variation in the timing of AMH measurement post-surgery in the literautre [9]; studies showing recovery of AMH at different time intervals [12]; and no agreed threshold at which a particular level of AMH decline is deemed clinically significant. Consensus on the types of ovarian reserve markers, frequency of measurement, and the degree of change within these markers that are clinically significant is needed. Future studies that control for different types of ovarian cysts (such as size, number), surgical techniques, and patient factors (such as age) are also required. Participant age is also an important determinant of AMH levels. Two of the participants in our case series were over 35 years of age and they had the lowest AMH levels at baseline. The impact of ovarian cystectomy in participants greater than 35 years therefore needs particular consideration in pre-operative counseling.

### Learning points

Ovarian cystectomy can result in ovarian injury and a decrease in ovarian reserve, which can be more pronounced with the use of electrosurgery for hemostasis.

The current literature suggests that there is a reduction in ovarian reserve following benign ovarian cystectomies overall, and this may recover within 1 year of surgery.

Individualized patient counseling, which takes into account their age, the cyst size, type, and number, should be considered pre-operatively.

### Conclusion

Our case series did not find a statistically significant change in AMH levels pre-operative to post-operative following laparoscopic dermoid cystectomy. The effect on fertility following dermoid cystectomies needs to be considered during counseling with patients and more data are required from future studies.

## Data Availability

The authors declare that data supporting the findings of this study are available within the article.

## Abbreviations

AMH: anti-Mullerian hormone
IVF: *in-vitro* fertilization
